# Preference for Shelters at Different Developmental Stages of Chinese Mitten Crab (*Eriocheir sinensis*)

**DOI:** 10.3390/ani12070918

**Published:** 2022-04-03

**Authors:** Chunbo Li, Chenchen Shen, Guangpeng Feng, Xiaorong Huang, Xincang Li

**Affiliations:** 1 East China Sea Fisheries Research Institute, Chinese Academy of Fishery Sciences, Shanghai 200090, China; li15896532342@163.com (C.L.); 18936152362@163.com (C.S.); hxr828@126.com (X.H.); lixin8687@163.com (X.L.); 2 College of Fisheries and Life Sciences, Shanghai Ocean University, Shanghai 200090, China

**Keywords:** shelter preference, *Eriocheir sinensis*, habitat selection, concealing behavior

## Abstract

**Simple Summary:**

Shelter is closely correlated with growth and development of aquatic crustaceans. Aquatic crustaceans often prefer to live in shelters to forage and avoid predators. In this study, Chinese Mitten Crabs (*Eriocheir sinensis*) at different developmental stages were selected to analyze preference for shelters. These results indicated that juvenile crabs had a significant preference for grass; button-sized crabs preferred to hide in mud; and the favorite shelters for parent crabs were rocks. Moreover, light, size, feeding habit, predation and molting were critical factors that affect the preference for shelters of *E. sinensis*.

**Abstract:**

A shelter is a good habitat for aquatic organisms, which could aid in avoiding cannibalism and facilitate predation. Chinese Mitten Crab (*Eriocheir sinensis*) is an important aquaculture species with troglodytism and nocturnal habit. To clarify the preference for shelters at different developmental stages of *E. sinensis*, different shelters (mud, sand, grass and rocks) were selected for comparison. These results indicated that juvenile crabs had a significant preference for grass; button-sized crabs preferred to hide in mud; and the favorite shelters for parent crabs were rocks, followed by mud. *E. sinensis* in three stages all showed concealing behavior. The concealing behavior of juvenile crabs was the most significant, followed by button-sized and parent crabs. Additionally, *E. sinensis* held a low hiding rate at night but a high hiding rate during the daytime due to nocturnal habits. These findings will help to better understand the habits of *E. sinensis* and provide a reference for resource restoration, habitat construction and the restoration of *E. sinensis*.

## 1. Introduction

Chinese mitten crab, *Eriocheir sinensis* (Crustacea, Decapoda, Varunidae), is an important aquaculture species with high production and economic values, which is also a representative crab species in China [1]. Meanwhile, *E. sinensis* is also an invasive species in Europe and America, which is notorious due to the destructive nature towards native biodiversity and the banks of rivers [2,3]. *E. sinensis* belongs to a migratory crustacean group that prefers to live in caves along rivers and lakes [4]. *E. sinensis* has several developmental stages, including zygote, zoea, megalopa, juvenile crab, young crab (button-sized) and adult crab [1]. *E. sinensis* needs to molt several times from zoea to adult, and molting occurs about five times in juvenile, button-sized and adult crabs, respectively, but most crabs are vulnerable to other individuals during the molting period [5,6]. In addition to the aggressive instinct of crabs, the lack of suitable shelters for molting in the environment is a reason for being attacked [7]. Studies have shown that crabs might molt less, even stop molting, and lose weight when the living conditions were unsuitable [8,9]. Therefore, a suitable shelter with high safety and abundant food is very important for the growth and development of crabs.

A shelter is a good habitat for aquatic organisms, which could help in avoiding cannibalism and facilitate predation [10]. Shelters could reduce the individual density in the water environment, and the low individual density promoting the growth of crustaceans has already been observed in *Fenneropenaeus chinensis*, *Paralithodes camtschaticus* and *Macrobrachium rosenbergii* [11,12,13]. Many field observations have found that appropriate shelters could reduce the probability of intraspecific aggression, provide food source and improve the survival rate of aquatic animals. For example, biological concealment, such as *Vallisneria natans* and *Myriophyllum spicatum*, can not only contribute to reducing direct contact between species and avoiding natural enemies but also be a food source for *M. rosenbergii*, which was consistent with the function of aquatic plants for juvenile crabs [14]. Similarly, submerged plants in ponds might benefit *E. sinensis* in the survival, growth and development, and the survival rate of juvenile *Callinectes sapidus* in vegetated habitat was significantly higher than that in unvegetated habitats [15,16]. The three-dimensional artificial floating wetlands could provide new habitats, which form a stable environment for nekton and increase the habitat available to juvenile fish [17]. Meanwhile, abiological substances, such as rocks, mud, plastic box and waste tire, are also often used as shelters by aquatic animals. Rocks are often utilized as shelters for holothurian to complete the aestivation and hibernation [18]. *Scylla paramamosain* could improve the survival and growth rates by using perforated brick, sand grains and clam shell as shelters [19]. Moreover, polyvinylchloride tubes could also be used as shelters for *Salvelinus alpines* to improve yields [20]. The shelter made by half of a plastic frame could effectively shorten the molting period and improve the weight growth rate of *Portunus trituberculatus* after molting [21]. Blue crabs were protected from predators in natural waters by seaweed, shells and filamentous algae [22]. Shelters also helped megalopa and juvenile crabs to maintain spatial position and avoid being driven away from favorable habitats by swift currents [23].

In the recent years, with the implementation of some water-related projects and the changes of water environment in the main stream of the Yangtze River, the habitat of *E. sinensis* has been severely damaged [24,25]. The loss of vegetation, sediment reduction and lack of rocks in the water environment had a great negative impact on the normal life of *E. sinensis*. As a consequence, in order to provide a reference for resource restoration, habitat construction and restoration of *E. sinensis*, mud, sand, grass and rocks were selected as shelters for comparison according to the habits of *E. sinensis*. The concealing behavior and preference for shelters of *E. sinensis* at different development stages were focused to select the most suitable shelter in this study.

## 2. Materials and Methods

### 2.1. Animal Collection and Source of Shelters

A total of 300 stage-3 juvenile crabs with the average body weight of (0.31 ± 0.15) g, 300 button-sized crabs with the average body weight of (12.23 ± 3.68) g, and 300 parent crabs with the average body weight of (111.03 ± 17.66) g, which had been mature, were selected from Gaoyou Lake, Jiangsu Province. During the acclimation period, crabs were fed with live razor clams at 2–3% of body weight of crabs every night. After a week, healthy and non-mutilated juvenile, button-sized and parent *E. sinensis* were selected for the experiment.

**Figure 1:**
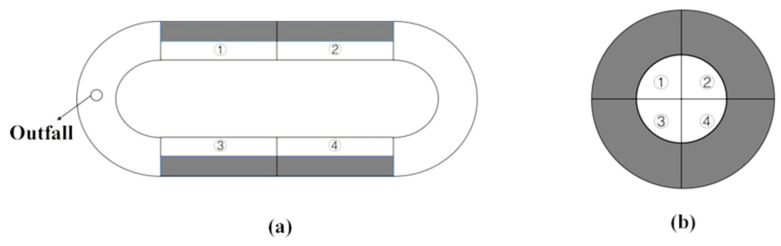
Different experimental devices for different development stages of *Eriocheir sinensis* ((**a**): the experimental device of parent crabs; (**b**): the experimental devices of juvenile and button-sized crabs). The numbers 1–4 represented four experimental areas including black areas and blank areas. Four black areas represented the location of four kinds of shelters respectively.

Mud, sand, grass and rocks were selected as shelters. Unlike the substrate, mud and sand were made into burrows for crabs to inhabit; rocks were piled up to form caves, and grass was normally planted in small amounts of soil. The mud was taken from the Yangtze estuary, which was dried and then ground to powder, and water was added to make mud stick together to simulate caves installed in the experimental device. The sand was washed to remove debris and then dried to simulate caves installed in the experimental device. Rocks with a surface area that was larger than the experimental crabs were used and piled up to form caves as shelters in the experimental device, and the rock’s surfaces were cleaned. Grass originating from aquaculture farms was washed and planted in small amounts of soil to hold the experimental device. Four kinds of shelters were, respectively, placed in each black part of the experimental devices (Figure 1).

### 2.2. Experimental Design and Procedure

Due to the different development stages of *E. sinensis*, three different experimental devices were designed. The experiments of juvenile crabs and adult mitten crabs were carried out in different experimental devices, respectively, without mutual interference. The experimental device of parent crabs was an annular tank with the length of 5 m, width of 2 m and height of 0.5 m. Both ends are semicircular with a radius of 1 m, and the middle is rectangular and measured 3 m long and 0.5 m wide (Figure 1a). The experimental devices of juvenile and button-sized crabs were cylindrical culture tanks (made of fiberglass) with a diameter of 0.6 m and 1 m, respectively, and the bottom was evenly divided into four parts (Figure 1b). The area in each black part where a kind of shelter was placed was the same as the blank area. Two infrared cameras were installed above the migration trough to ensure that the entire migration trough could be monitored. In order to avoid interference, the entire test area was surrounded by a curtain and isolated from the surrounding area. Observation and recording were carried out by an infrared camera.

Concealing behavior experiments: Before experiments, crabs were removed from tanks and dried with a dry towel. A white reflective film was affixed to carapaces for observation. Crabs were released in the center of the experimental device and acclimated for 10 min. The concealing behavior experiments were carried out during the daytime and at night. The infrared cameras were used to observe whether crabs were hidden in the shelters. The hiding ratio referred to the proportion of crabs in the shelters, and the hiding ratio was used to judge whether crabs showed concealing behavior.

Selection of shelters experiments: The previous treatment was the same as the concealing behavior experiments. The experiments were conducted during the daytime and at night. The parent crab group took individuals as an experimental unit. In the experiment, every parent crab was released in the center of the experimental device for 10 min, and then the infrared cameras were used to record the distribution of parent crabs for 5 min (residence time in each shelter). Parent crabs were divided into four groups with eight crabs in each group, and each experiment was repeated three times. Before the beginning of each experiment, the positions of four shelters were randomly changed to prevent the preference of *E. sinensis* for a certain location in the tank from affecting experimental results. The experiment method of button-sized crabs was the same as parent crabs. However, due to the strong movement ability of the button-sized crab, the adaptation time was extended to 20 min. The button-sized crabs were divided into four groups, with four crabs in each group, and each experiment was repeated three times. Given that the individual juvenile crabs were too small to be observed with cameras, the population experiment was carried out for juvenile crabs. Juvenile crabs were divided into four groups with thirty crabs in each group, and each experiment was repeated three times. The number of experimental crabs in each shelter was recorded. All crabs were alive after experiments, and the survival rate was 100%.

### 2.3. Data Acquisition and Statistical Analysis

One-way ANOVA was used to determine the significant differences in preference for shelters of *E. sinensis* (*p* < 0.05). Duncan’s multiple range test was performed to analyze the differences between shelters. The significant differences between groups were tested by the independent samples *t*-test. All data were analyzed using SPSS 24.0 (IBM, Armonk, NY, USA), and these results were presented as Mean ± SD (standard deviation of the mean).

## 3. Results

### 3.1. Concealing Behavior of E. sinensis in Different Developmental Stages

**Figure 2:**
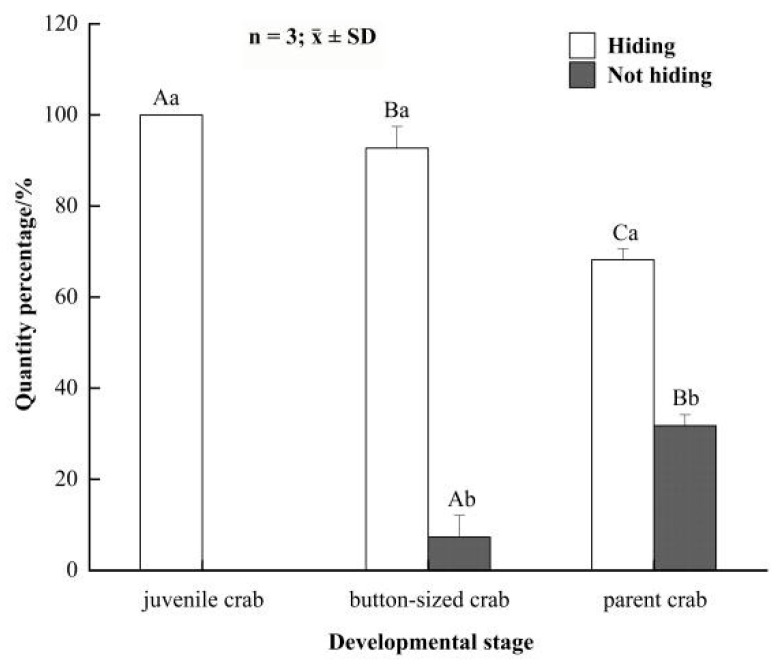
The hiding rate of *Eriocheir sinensis* in different developmental stages. Bars with same letters on the top indicate they have insignificant differences (*p* > 0.05), and bars with different letters on the top indicate they have significant differences (*p* < 0.05). Capital letters indicate the significance of hiding rates in different developmental stages, and lowercase letters indicate the significance of hiding and not hiding in the same stage.

The hiding rate was 100% for juvenile crabs, and the hiding rate was (92.71 ± 4.77)% for button-sized crabs, showing a significant difference between juvenile and button-sized crabs (Figure 2). For parent crabs, the hiding rate (68.23 ± 2.39)% indicated a significant difference between hiding and not hiding (Figure 2). Therefore, the concealing behavior of juvenile crabs was the most significant, followed by button-sized and parent crabs.

### 3.2. Differences in Concealing Behavior of E. sinensis between Daytime and Night

**Figure 3:**
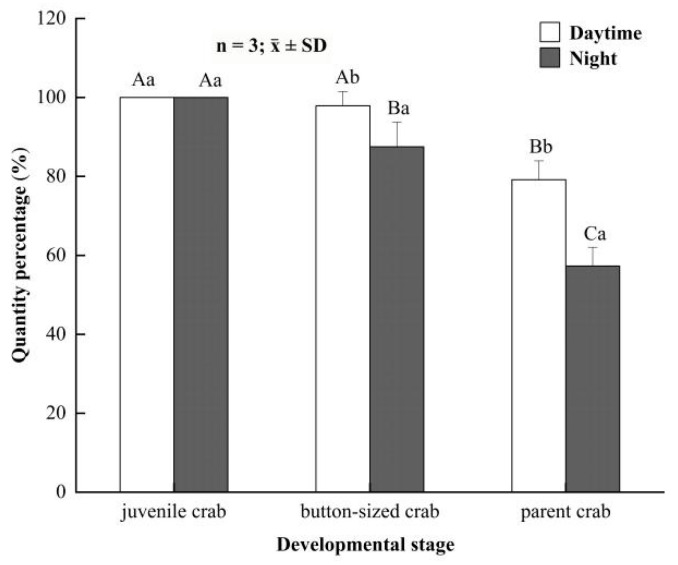
The hiding rate of *Eriocheir sinensis* in different developmental stages between daytime and night. Bars with same letters on the top indicate they have insignificant differences (*p* > 0.05), and bars with different letters on the top indicate they have significant differences (*p* < 0.05). Capital letters indicate the significance of hiding rates in different developmental stages, and lowercase letters indicate the significance of hiding and not hiding in the same stage.

For juvenile crabs, the hiding rate (100%) showed no significant difference between night and daytime (Figure 3). In daytime, the hiding rate of button-sized crabs was (97.92 ± 3.61)%, which was significantly higher than that at night (*p* < 0.05). Parent crabs yielded analogous results in our study. The hiding rate was (79.17 ± 4.77)% in daytime, which was also significantly higher than that at night (57.29 ± 4.77)%.

### 3.3. Preference for Shelters in the Juvenile Crabs

**Figure 4:**
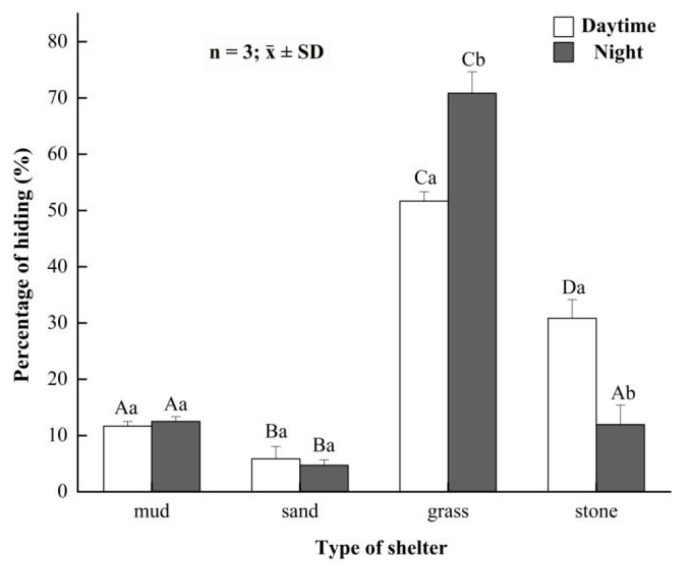
The hiding rate of juvenile *Eriocheir sinensis* in different shelters between daytime and night. Bars with same letters on the top indicate they have insignificant differences (*p* > 0.05), and bars with different letters on the top indicate they have significant differences (*p* < 0.05). Capital letters indicate the significance of hiding rates in different developmental stages, and lowercase letters indicate the significance of hiding and not hiding in the same stage.

In daytime, the percentages of time that juvenile crabs hid in mud, sand, grass and rocks were (11.67 ± 0.84)%, (5.83 ± 2.20)%, (51.67 ± 1.67)% and (30.83 ± 3.33)%, respectively (Figure 4). Juvenile crabs spent the longest time hiding in grass in daytime, followed by rocks, mud and sand, showing significant differences in four shelters (*p* < 0.05). At night, the percentages of time that juvenile crabs hid in mud, sand, grass and rocks were (12.5 ± 0.83)%, (4.72 ± 0.96)%, (70.83 ± 3.82)% and (11.94 ± 3.47)%, respectively (Figure 4). Juvenile crabs spent the longest time hiding in grass at night, followed by mud, rocks and sand, showing significant differences among four different kinds of shelters (*p* < 0.05). Moreover, juvenile crabs spent more time staying in rocks during daytime than night, and juvenile crabs spent more time staying in grass during night than daytime, indicating significant differences between night and daytime (*p* < 0.05). However, mud and sand had no significant difference between night and daytime (*p* > 0.05).

### 3.4. Preference for Shelters in the Button-Sized Crabs

**Figure 5:**
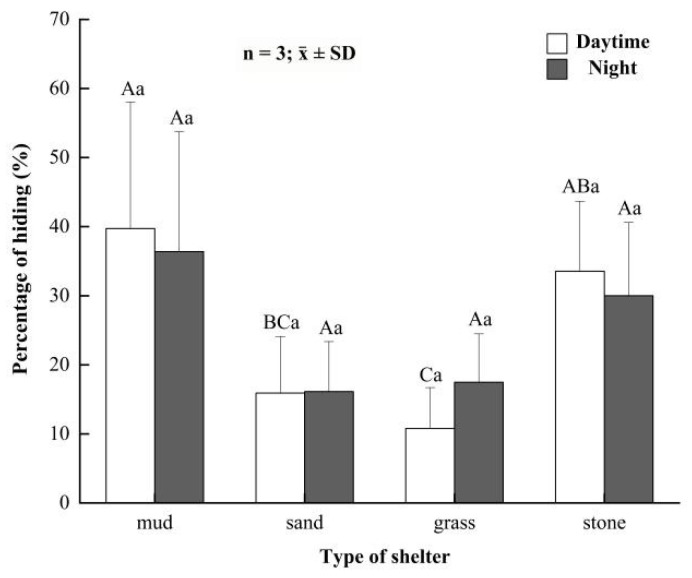
The hiding rate of button-sized *Eriocheir sinensis* in different shelters between daytime and night. Bars with same letters on the top indicate they have insignificant differences (*p* > 0.05), and bars with different letters on the top indicate they have significant differences (*p* < 0.05). Capital letters indicate the significance of hiding rates in different developmental stages, and lowercase letters indicate the significance of hiding and not hiding in the same stage.

In daytime, the percentages of time that button-sized crabs hid in mud, sand, grass and rocks were (39.74 ± 18.28)%, (15.92 ± 8.16)%, (10.78 ± 5.93)% and (33.55 ± 10.13)%, respectively (Figure 5). Parent crabs spent significantly more time hiding in mud than in sand and grass at night (*p* < 0.05), but no significant difference was observed in mud and rocks (*p* > 0.05). At night, the percentages of time that the button-sized crabs hid in mud, sand, grass and rocks were (36.39 ± 17.34)%, (16.14 ± 7.22)%, (17.47 ± 7.01)% and (30.01 ± 10.61)%, respectively (Figure 5). The hidden time of parent crabs showed no significant difference among four kinds of shelters (*p* > 0.05). Additionally, the hidden time of parent crabs had insignificant differences between night and daytime.

### 3.5. Preference for Shelters in the Parent Crabs

**Figure 6:**
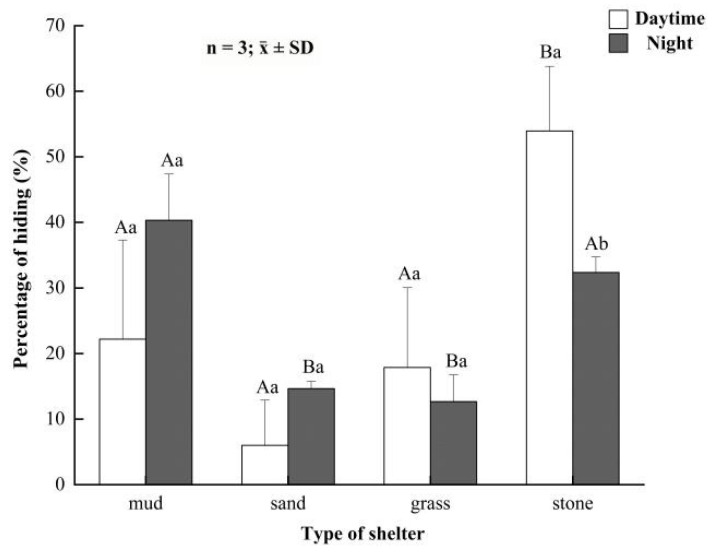
The hiding rate of parent *Eriocheir sinensis* in different shelters between daytime and night. Bars with same letters on the top indicate they have insignificant differences (*p* > 0.05), and bars with different letters on the top indicate they have significant differences (*p* < 0.05). Capital letters indicate the significance of hiding rates in different developmental stages, and lowercase letters indicate the significance of hiding and not hiding in the same stage.

In daytime, the percentages of time that parent crabs hid in mud, sand, grass and rocks were (22.18 ± 15.12)%, (6.00 ± 6.89)%, (17.87 ± 12.19)% and (53.94 ± 9.87)%, respectively (Figure 4). The hiding time of parent crabs was significantly more in rocks than other shelters in daytime (*p* < 0.05). At night, the percentages of time that parent crabs hid in mud, sand, grass and rocks were (40.33 ± 7.03)%, (14.64 ± 1.11)%, (12.66 ± 4.11)% and (32.36 ± 2.39)%, respectively (Figure 6). The hidden time of parent crabs in mud and rocks was significantly higher than that in sand and grass at night (*p* < 0.05), but there was no significant difference between mud and rocks (*p* > 0.05).

## 4. Discussion

The transition stage is the weakest phase for aquatic crustaceans as they are seriously vulnerable to predation and invasion from other aquatic animals [26]. Thus, selecting suitable shelters is necessary for crustaceans. Moreover, previous studies have reported that stereo artificial floating wetlands could enhance the habitat of juvenile fish in the degraded habitats along the estuary, and they were conducive to creating spawning grounds for phytophilous fish in urban rivers, suggesting that suitable shelters contribute to the growth and reproduction of aquatic animals [27,28]. At present, most research studies on shelters had focused on the effects on growth indicators, reproduction and spatial distribution [29,30]. Nevertheless, research on preference for shelters of aquatic crustaceans, especially at different developmental stages, remains limited. In the natural environment, juvenile crabs living in lakes often use aquatic plant or big rocks as hiding objects to hide themselves from harm, and mud is often washed by the current and becomes moist, which is easy to dig holes for crabs [31]. Hence, mud, sand, grass and rocks were selected as shelters to study the concealing behavior and preference for shelters of *E. sinensis* at different development stages in this study.

The juvenile, button-sized and parent *E. sinensis* all exhibited concealing behavior. Compared to button-sized and parent crabs, juvenile *E. sinensis* preferred to hide. This phenomenon might be caused by resistance to the light of juvenile *E. sinensis*. After metazoa developed into juvenile crabs, *E. sinensis* changed to benthic life, and juvenile crabs preferred habitats with weak light [32]. Then, shelters could provide dark environments for crabs to survive. As they were growing, juvenile crabs grew into button-sized crabs and eventually into parent crabs. Compared with parent crabs, juvenile and button-sized crabs are vulnerable to predation from aquatic animals. Previous studies have shown that shelters could induce significant improvements in the survival rate of juvenile *Scylla serrara*, and both survival and growth rates of mud fish increased in the shelters (grass and composite boards) [33,34]. Thus, shelters could provide a natural protective barrier for young crabs to reduce casualties. Finally, concealing behaviors could greatly reduce the movement to save more energy for body growth or molting [35,36]. Thereby, juvenile *E. sinensis* preferred to hide in the shelters for better growth and higher survival rates.

Juvenile crabs had a significant preference for shelter (grass) in this study, which might be related to predation and anti-predation. The main food source of juvenile crabs is aquatic plants in nature, and grass could provide energy for juvenile crabs [37]. Then, crabs lacked activity and self-protection ability, and the survival rate was often low in the early stage of life [38,39]. Aquatic plants could provide suitable living environments for juvenile crabs to attach and avoid predators. As they were growing, juvenile crabs grew into button-sized crabs. Due to stage specificity, button-sized crabs showed no obvious preference for four shelters. The button-sized crabs need to molt several times before growing into adult crabs, and molting is a complex process that needs more energy to generate fresh shell or maintain metabolism [40]. Therefore, button-sized crabs have to move around to find more food to replenish energy before molting. Despite insignificant differences, button-sized crabs preferred to hide in the mud compared to other shelters in this study. As they were growing, button-sized crabs grew into parent crabs. For parent crabs, the favorite shelters were rocks, followed by mud, which agreed with the report by Yonezawa [41]. The parent crabs mainly appear in coastal estuaries and islands, and the lifestyle of *E. sinensis* is troglodytism in riparian beach land [42]. Both rocks and mud could provide parent crabs with caves. Parent crabs are larger than juvenile and button-sized crabs, so rocks and mud are more conducive for hiding for parent crabs. Furthermore, adult crabs tend to be carnivorous, so shelters (rocks and mud) could assist parent crabs to hunt other aquatic animals, and caves could protect parent crabs from predators such as herons, gulls and cranes [43,44]. Taken together, the preferences for shelters of *E. sinensis* were diverse at different developmental stages due to size restriction, feeding habit, predation and molting. These results could provide a reference for resource restoration, habitat construction and the restoration of *E. sinensis* in China. Moreover, rocks or grass-covered habitats were vulnerable to invasion by crabs as shelters, which could lead to population expansion. Hence, removing these shelters might contribute to catching and removing crabs in Europe and America.

Previous reports have confirmed that shrimps and crabs have the habit of hiding during daytime and foraging at night [45]. *E. sinensis* has an obvious feeding rhythm, and the best feeding time is at night [46]. In current study, button-sized and parent crabs were better at hiding during daytime than night. Our study showed that *E. sinensis* held a low hiding rate at night but a high hiding rate in daytime, which was consistent with the nocturnal habit of crabs [47]. Moreover, the concealing behavior of *E. sinensis* between daytime and night was similar to some aquatic animals. The hiding rate of *Penaeus japonicus* in daytime was higher than night, resulting in a 36% average decrease in cannibalism (*p* < 0.05) [48]. *Panulirus stimpsoni* was more active at night, and *P. japonicus* showed a nocturnal feeding pattern [49,50]. The climbing density and climbing proportion of *Chiromantes neglectum* in reed at night were significantly higher than those in daytime [51]. The preferences for crevices in *Glyptosternum maculatum* larvae and juveniles were significantly higher in daytime than night [52]. The preference for concealing behavior in daytime of *E. sinensis* could provide more references for the study of feeding times.

## 5. Conclusions

In summary, the preferences for shelters of *E. sinensis* were diverse at different developmental stages: juvenile crabs had an obvious preference for grass; button-sized crabs preferred to hide in the mud; and the favorite shelters of parent crabs were rocks. In our study, juvenile, button-sized and parent *E. sinensis* all exhibited concealing behavior, which occurred mainly during daytime. Moreover, light, size restriction, feeding habit, predation and molting were main factors that affect the preference for shelters at different developmental stages of *E. sinensis*. In order to effectively increase the resources of *E. sinensis* in China, *E. sinensis* at different developmental stages should be raised in areas with different shelters (i.e., grass, rocks and mud) according to the preference for shelters to improve the survival rate. Moreover, America and Europe could regularly clean rocks and grass on the banks of rivers to reduce crab infestation.

## Data Availability

The data presented in this study are available in the article. Further information is available upon request from the corresponding author.
